# A Novel Nanocomposite Based on Triazine Based Covalent Organic Polymer Blended with Porous g-C_3_N_4_ for Photo Catalytic Dye Degradation of Rose Bengal and Fast Green

**DOI:** 10.3390/molecules27217168

**Published:** 2022-10-23

**Authors:** Nachimuthu Venkatesh, Govindhasamy Murugadoss, Abdul Azeez Ashif Mohamed, Manavalan Rajesh Kumar, Shaik Gouse Peera, Pachagounder Sakthivel

**Affiliations:** 1 Department of Nanoscience and Technology, Bharathiar University, Coimbatore 641046, Tamil Nadu, India; 2 Centre for Nanoscience and Nanotechnology, Sathyabama Institute of Science and Technology, Chennai 600119, Tamil Nadu, India; 3 Institute of Natural Science and Mathematics, Ural Federal University, 620002 Yekaterinburg, Russia; 4 Department of Environmental Science, Keimyung University, Dalseo-gu, Daegu 42601, Korea

**Keywords:** graphitic carbon nitride, organic porous polymer, metal free nano composite, toxic dye, photocatalytic, visible sunlight

## Abstract

Metal free visible light active photocatalysts of covalent organic polymers (COPs) and polymeric graphitic carbon nitride (g-C_3_N_4_) are interesting porous catalysts that have enormous potential for application in organic pollutant degradation. Imine condensation for COPs, and thermal condensation for g-C_3_N_4_ were used to produce the catalysts. FT-IR, Raman, NMR, UV-Vis Spectroscopy, X-ray diffraction, and scanning electron microscopy studies were used to investigate the structural, optical, and morphological features of the metal free catalysts. We have constructed COPs with a π-electron deficient (Lewis acidic) triazine core and π -electron rich (Lewis basic) naphthalene and anthraquinone rings coupled by -O and -N donors in this study. Furthermore, the prepared Bulk-g-C_3_N_4_ (B-GCN) was converted to porous g-C_3_N_4_ (P-GCN) using a chemical oxidation process, and the generated P-GCN was efficiently mixed with the COP to create a novel nanocomposite for photocatalytic application. Using the anthraquinone-based COP and P-GCN (1:1 ratio, PA-GCN) catalyst, the highest photodegradation efficiencies for the polymeric graphitic carbon nitride of 88.2% and 82.3% were achieved using the Fast green (FG) and Rose bengal (RB) dyes, respectively. The rate constant values of 0.032 and 0.024/min were determined for FG and RB degradation, respectively. Higher activity may be related to the incorporation of COP and PA-GCN, which act significantly well in higher visible light absorption, have superior reactive oxygen generation (ROS), and demonstrate an excellent pollutant–catalyst interaction.

## 1. Introduction

The ecosystem is currently considered to be critically harmed by surface water adulteration, water desertion, and a lack of drinking water. The direct discharge of numerous organic poisons from the pharmaceutical, chemical, and textile industries into the environment and water bodies is the primary source of water contamination. As a result, the active process of photocatalysis, which may recover organic contaminants, has enormous potential for removing harmful substances from water. Semiconductor-based photocatalysis has emerged with inestimable superiority because it is considered to be an economic, renewable, clean and safe technology. It requires only the inexhaustible solar light as a driving force and a suitable semiconductor as a photocatalyst, to conduct catalytic reactions for a variety of applications, such as hydrogen production from water splitting, CO_2_ reduction into hydrocarbon fuels, decomposition and mineralization of organic pollutants, selective organic synthesis, and even disinfection of bacteria. Metal free photocatalysts always possess good separation of electron−hole pairs, fair response to visible light, and a suitable and tunable band gap for maximally utilizing sunlight energy. Graphitic carbon nitride (g-C_3_N_4_) is widely used in the field of photocatalyst degradation. There are many types of photocatalytic materials: zero-dimensional granular photocatalytic material, one-dimensional linear photocatalytic material, two-dimensional layered photocatalytic material, and three-dimensional bulk photocatalytic material. Among these, g-C_3_N_4_ has attracted extensive attention in recent years due to its excellent performance, with attributes such as highly efficient photocatalytic hydrogen generation, water oxidation, carbon dioxide reduction, organic pollutant degradation, and artificial photosynthesis.

Notably, researchers are fascinated by g-C_3_N_4_, a 2D π-conjugated metal free polymeric semiconductor with a mild band gap (2.7 eV), and a visible light absorption capacity of ~43% of the solar spectrum. It has emerged as an attractive material for various scientific applications due to its excellent thermal stability, chemical stability, proper electronic band structure, nontoxicity, low cost, good stability, and easy preparation [1]. g-C_3_N_4_ has been considered the most stable form under ambient conditions compared to its counterpart allotropes such as α-C_3_N_4_ [2], β-C_3_N_4_ [3], cubic C_3_N_4_, pseudo cubic C_3_N_4_, g-h-triazine, and g-o-triazine. g-C_3_N_4_ comprises of stacked two-dimensional (2D) sheets of heptazine interconnected via tertiary amines [4]. Structurally similar to graphite, g-C_3_N_4_ contains strong covalent C–N bonds instead of C–C bonds in each layer, while the layers are bound by van der Waals forces [5]. The presence of sp^2^ hybridized carbon and nitrogen, pertaining the *π*-conjugated electronic structures reported onset absorption at 460 nm. The g-C_3_N_4_ contains a tunable band gap within the controllable lowest unoccupied molecular orbital (LUMO), and the highest occupied molecular orbital (HOMO). This can remarkably affect the photoelectronic performance of g-C_3_N_4_ [6]. It is potentially a great photocatalyst to degrade organic pollutants into H_2_O and CO_2_ due to its unique properties [7].

The g-C_3_N_4_ and g-C_3_N_4_ heterostructures have emerged in use across a number of fields such as sensors, supercapacitors, adsorption, organic synthesis, hydrogen evolution from water splitting, photocatalytic degradation of microbials, organic and inorganic pollutants, bioimaging, oxygen reduction reactions, lithium-ion batteries, biofuel cells, lubrication, photo–chemo combination therapy, as an additive in FO membranes, etc. Many facile methods can be employed for the synthesis of g-C_3_N_4_, such as chemical vapor deposition (CVD), solvothermal synthesis, solid-state reaction, and thermal decomposition [8]. Particularly, Bulk g-C_3_N_4_ powder can be prepared by the thermal polycondensation of low-cost nitrogen containing organic precursors, such as urea, thiourea, melamine [9], cyanamide [10], dicyandiamide [11], guanidine hydrochloride [12], and others [13,14,15,16,17]. The pure bulk g-C_3_N_4_ prepared by this method suffers from several shortcomings, including small surface area, high surface inertia, low utilization of solar energy, low carrier mobility, fast recombination rate [18], and low conductivity [19]. However, the material can be modified to overcome the above weaknesses. Enhancing the photocatalytic properties is reported by structural modification through the addition of foreign substances [20,21], co-polymerization [22,23], or incorporation of carbonaceous and metal deposits [24,25]. In structural modification, porous materials are prepared by templating methods that consider controlled surface areas, large active sites, morphology, and pore diameter [26]. Structure and morphology are varied by varying temperature, time, and precursors. Similarly, covalent organic frameworks (COFs) have recently gathered increasing attention. COFs are crystalline polymers constructed from a large variety of organic building blocks via covalent bonds. Covalent Organic Frameworks (COFs) are an emerging class of porous, irregularly extended 2D and 3D polymers synthesized through polymerization and crystallization of monomeric building units [27,28]. COFs are distinct from molecular compounds and linear polymers because they organize monomers into networks with well-defined pores and high internal surface areas [29,30]. COFs are a class of crystalline polymers bearing micro porous or mesoporous pores, which are built by the combination of light elements (eg., B, C, Si, N, O) through the strong covalent bonds. COFs have plenty of unique properties such as ordered channels, large surface areas, highly tunable porosity, optional building blocks, predictable and stable structures, and abundant functional groups, resulting in wide applications in many fields. Some organic semiconductor photocatalysts’ optoelectronic capabilities may be modified by manipulating the alignment ordering of donors and acceptors, as well as the production of symmetrical or asymmetrical structure. Hence, COFs can provide hopeful platforms for various applications such as heterogeneous catalyst semiconductors [31], sensors [32], gas storage [33], catalysis [34,35,36], opto-electronic devices [27,37,38], energy conversion, and storage devices [39].

COFs can be divided into two dimensional (2D) and three dimensional (3D) structures based on dimensions. In 2D COFs, covalent bonds only exist in conjugated 2D sheets while only weak interactions (such as π-π stacking, hydrogen bonds and van der Waals’ forces) are present in interlayers. In contrast, the whole skeletons of 3D COFs are connected by covalent bonds. Scientists are focused on developing porous COFs due to their multi chemical functionality. Pores on the COFs have unique compensations in binding and separation; they can be synthesized using imine, boronate ester, borazine, hydrazine, boroxine, borosilicate, triazine, squaraine, and azodioxide linkages to structure blocks that are linked by covalent bonds [34]. According to reticular chemistry theory, we know that COF materials could be constructed from rigid organic building units with varying structural configurations. The chemical and physical properties of COFs can be easily tuned through a selection of building blocks and linkage motifs.

Herein, we aimed to construct a catalyst with the highest light absorption and greatest reactive oxygen radical generation. Hence, the g-C_3_N_4_ with COF composite was developed to generate several reactive species. Photocatalytic dye degradation against the model organic pollutants of Fast green (FG) and Rose bengal (RB) molecules were studied.

## 2. Materials and Synthesis Procedures

### 2.1. Materials

Cyanuric chloride, 4-hydroxybenzaldehyde, Sodium carbonate, 1,5-diaminonaphthalene, and 2,6-aminoanthraquinone were analytical reagents and obtained from Sigma-Aldrich Bangalore, Karnataka, India, HI Media Company, Thane west, Maharashtra, India. supplied Fast green and Rose bengal, the SDFCL firm supplied the absolute ethanol and p-toluene sulphonic acid. Preparation of nanocomposites (NCs) and photocatalytic experiments were carried out with double-distilled water (DI). The glassware was cleaned with aqua regia before being rinsed with DI water.

### 2.2. Synthesis of Triazine Based Monomer and Polymer

#### 2.2.1. Synthesis of Triazine Based Triarylaldehyde (Tripod)

**Figure 1:**
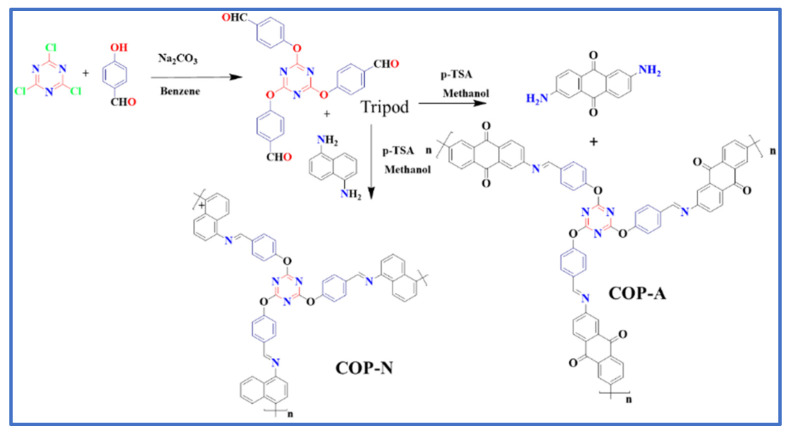
Synthesis route of covalent organic polymer of COP-N and COP-A.

4-hydroxy benzaldehyde (1.58 g, 13 mmol) and cyanuric chloride (5.99 g, 3.25 mmol) were added to a suspension of Na_2_CO_3_ (7g) in 50 mL of benzene. The mixture was refluxed for 20 h. Then, the reaction mixture was cooled and the solid was removed by extraction with hot AcOEt, the same washing procedure followed twice. Next, the extract was washed with 10% Na_2_CO_3_ solution two times followed by H_2_O once. Finally, the organic layer was dried over anhydrous MgSO_4_. The white powder was recrystallized from 20 mL of AcOEt to afford 3.76 g (64.9%) of a white fluffy precipitate and synthesis route of monomer shown in Figure 1. M.P-.172–174 °C, ^1^H-NMR (CDCl_3_, 400 MHz): δ 10.01 (s, 3H), 7.82 (d, 6H), 7.50 (d, 6H). FT-IR peaks (KBr pellet cm^−1^): 3387(w), 2911(w), 2859(w), 1712(s), 1602(m), 1556(vs), 1489(m), 1369(s), 1292(m), 1139(m), 837(m), 625(m), 503(w).

#### 2.2.2. Synthesis of Polymer of COP-N and COP-A

Compound 1 (Tripod) (1.5 g, 3.39 mmol) and 1,5 diaminonaphthalene (0.804g, 4.8 mmol) were dissolved in 15 mL of methanol. After that, the catalytic amount (0.01 g) of p- toluene sulphonic acid (p-PSA) was added in the above reaction mixture and it was kept at 65 °C for 48 h. Then, the mixture was poured into water for washing. After the filtration, the obtained residue was dried in a vacuum oven. The prepared triazine based polymer (COP-N) was not soluble in any organic solvent or water. Further, it was purified using Soxhlet extraction. Similarly, compound 1 reacted with 2,6 aminoanthraquinone (1.07 g 1.6 mmol) in the presence of p-PSA to give another triazine based polymer of COP-A and synthesis route of polymer shown in Figure 1. Yield: 56%. ^13^C SSNMR (500MHz): 199.3, 190.3, 169.6, 119.8, and 109.3 ppm for COP-N, 119.6, 180.6, 170.0, 150.4, 139.4, 120.5, 109.9, 90.0 and 80.0 for COP-A. FT-IR peaks (KBr pellet cm^−1^) COP-N: 3110(w), 1560(m), 1484(m), 1355(m), 1159(m), 837(w). COP-A: 3108(w), 1602(w), 1565(m), 1480(m), 1350(m), 1159(m), 830(m), 803(w).

#### 2.2.3. Synthesis of Bulk and Porous g-C_3_N_4_

The bulk g-C_3_N_4_ (B-GCN) sample was prepared by heating N-containing melamine precursors to 550 °C in a tubular furnace for 4 h. After the thermal condensation reaction, the resulting B-GCN sample was treated with a chemical oxidation process by the addition of 1 M of oxidizing agent of K_2_Cr_2_O_7_/H_2_SO_4_ solution and aged for 24 h. Then the above mixture was washed and dried at 120 °C under vacuum conditions. The prepared porous g-C_3_N_4_ (P-GCN) catalyst was used for photocatalytic activity.

#### 2.2.4. Synthesis of COP and g-C_3_N_4_ Based Nanocomposites

In methanol, a portion of the pre-synthesized COP-N and COP-A was thoroughly mixed with P-GCN (1:1 wt ratio) to generate a well-dispersed solution. Following methanol volatilization, the mixture was crushed into fine particles and heated to 250 °C for 2 h in a nitrogen environment. Finally, the acquired nanocomposites (PN-GCN and PA-GCN) were washed and dried to generate the final catalyst for photocatalytic activity.

### 2.3. Measurement

X-ray diffraction (XRD) analysis (Rigaku–Ultima IV) was utilized to determine the crystal structure of prepared B-GCN, P-GCN, COP-N, and COP-A. FE-SEM FEI (Quanta-250) and TEM (JEM 2100 F) were used to examine the morphology of the samples. The optical observations were carried out using an UV-Visible spectrophotometer (JASCO V-650) and Fourier transform infrared (FT-IR) analysis done at room temperature for analysis of functional groups presence on the surface of samples using a Bruker optic GmbH TENSOR-27 spectrometer. Confirmation of the polymers was studied using solid-state NMR analysis (Bruker, 500 MHz) and the sample’s vibrational phonon modes were investigated using Raman spectroscopy (Horiba Jobin-LabRam-HR, Chennai, Tamil Nadu, India.

### 2.4. Sun Light Active Photocatalytic Study of Fast Green (FG) and Rose Bengal (RB)

To study the photocatalytic dye degradation of the generated catalyst, the degradation dye of FG and RB (20 ppm, 50 mL) was evaluated in daylight. The dye solution was exposed to sunlight for 3 h. In this treatment, 2 mL of dye solution was removed from the treated solution container at fixed intervals. A total of 25 mg of the catalyst was used in the FG and RB dye solution. This dye solution was exposed to sunlight for 2 h, and a fixed amount of dye solution was collected at regular intervals for intensity measurement with a UV–Visible spectrophotometer.

The maximum absorbances (λ_max_) of FG and RB are 624 nm and 546 nm, respectively. The formula for calculating the degradation efficiency is,

Degradation efficiency (%) = (C_0_ − C)/C_0_ × 100 (1)

where C_0_ and C are the initial and variable intensities of the dye molecules, respectively. After the first cycle, the treated catalyst was centrifuged, washed with C_2_H_5_OH/H_2_O solution, and dried at 90 °C for the catalytic stability study. The stability and anti-pollutant properties of the regenerated catalyst were examined. Furthermore, the following 1st order kinetic research was carried out to identify the actual depolluting rate:

−ln(C/C_0_) = kt (2)

where k (min^−1^) is rate of reaction constant and t (min) is time. The photocatalytic study was carried out in bright sunlight from 11:30 a.m. to 2:00 p.m., and the typical sunlight intensity was measured with a Digital Lux meter. As a result, the average sunlight intensity was 0.95 × 10^5^ lux, computed at 30 min intervals.

## 3. Result and Discussion

### 3.1. Synthesis of Triazine Based Monomer and Polymer

**Figure 2:**
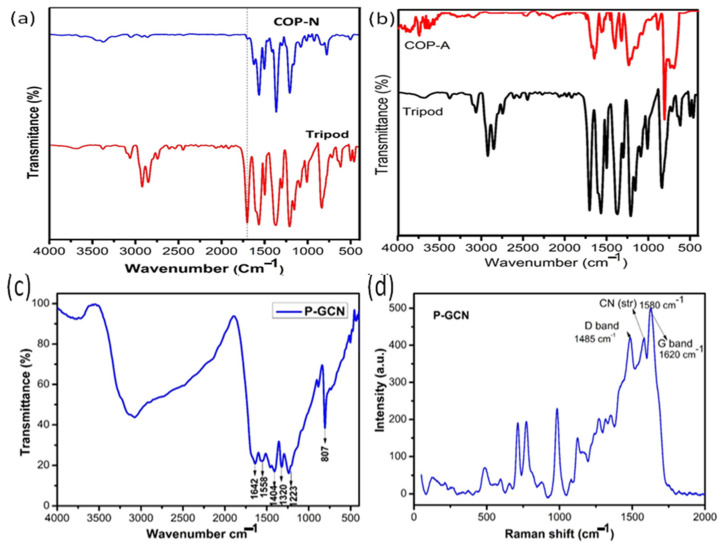
FT-IR spectra of (**a**) COP-N, (**b**) COP-A, (**c**) P-GCN and (**d**) Raman spectrum of P-GCN.

The technique of FT-IR analysis is important and widely utilized for determining the functional groups of prepared metal free catalyst such as the triazine based organic polymers of COP-N, COP-A, and porous g-C_3_N_4_ (P-GCN) (Figure 2a–c). The main functional group of triarylaldehyde (Figure 2a red line & Figure 2b black line) was confirmed by the characteristic peaks of C=O, and the aromatic group of C=C, which appeared at 1712 cm^−1^ and 1602–1556 cm^−1^, respectively. We concentrated on the functionalized NH_2_ and C=O groups to make them easier to identify. After the polycondensation reaction, the results (Figure 2a blue line & Figure 2b red line) confirmed the formation of a polymer due to the disappearance of NH_2_ and C=O, and the appearance of C=N stretching vibrations at 1602–1565cm^−1^. However, there is the presence of a weak peak at 3110 cm^−1^ of the Schiff base polycondensation reaction, due to the terminal NH_2_ and C=O groups on the surface.

Likewise, as the FT-IR spectra of P-GCN depicts in Figure 2c, a characteristic signal appeared between the 3600 to 3400 cm^−1^ range for the N-H stretching vibrations, and at 1800 cm^−1^ for the trigonal C-N(-C)-C or bridging C-NH-C center core unit of the triazine. The chemically oxidized triazine derivative shows the important peaks at 1404 and 1223 cm^−1^ represent the C-O and C-O-C functional groups, respectively. As a consequence, we assumed that the changes in IR peaks were produced by the increased strength of C-N covalent bonds generated by the electrophilic impact of oxygen atoms in new C-O bonds close to C-N. The Raman spectrum of the P-GCN is shown in Figure 2d. The existence of oxygen species in porous materials is also confirmed using Raman spectroscopy. Particularly, the peaks at 1485 and 1620 cm^−1^ represent the D and G bands of the P-GCN, respectively. In addition, another important peak at 1580 cm^−1^ shows the C-N stretching vibrations.

**Figure 3:**
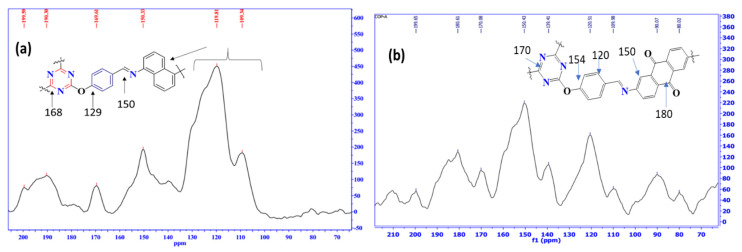
^13^C SSNMR spectra of triazine based polymer of the (**a**) COP-N and (**b**) COP-A.

Additionally, the ^13^C SSNMR analysis of COP-N and COP-A are illustrated in Figure 3. This massive peak of around 170 ppm clearly indicates the triazine core. The characteristic peaks at 129 and 150 ppm represent the C-O and C-N bonds, respectively. Likewise, both the COP-N and COP-A polymers show a significant imine bond signal around at 168 ppm, which indicate the C=N bond; no peak could be found in the aliphatic region, indicating that no tertiary carbon was present in the framework. Further, the COP-A exhibits important C=O bond signals at 180 ppm and 190 ppm.

### 3.2. XRD Analysis

**Figure 4:**
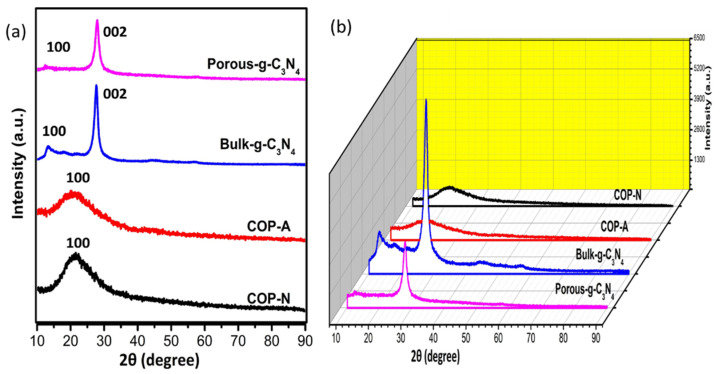
XRD spectra (**a**) 2D and (**b**)3D view of B-GCN, P-GCN, COP-N and COP-A. The yelloe are indicates the intensity (a.u.).

The powder XRD patterns of the 2D and 3D views of the prepared photocatalysts are shown in Figure 4a,b. Evidently, the COP-N and COP-A exhibit a diffraction peak at 22.3° indexed to the (1 0 0) crystal plane, which illustrates the π-π stacking between two layers and the periodicity of the covalent framework. Further, according to the literature, the wide peaks demonstrate the polymer’s amorphous nature. Likewise, the B-GCN and P-GCN clearly display two characteristic diffraction peaks at 12.84° and 28.24°, corresponding to the (1 0 0) and (0 0 2) crystal planes of hexagonal g-C_3_N_4_ (JCPDS 87-1526), respectively. The (0 0 2) peak, which corresponds to the interlayer stacking of g-C_3_N_4_, displays a slight shifting towards a higher angle (29.03°), showing that the acidification process flattens the g-C_3_N_4_ layers and narrows the interlayer spacing in P-GCN. In comparison to B-GCN, the overall intensity of P-GCN is lower in the 3D profile. The π-π stacking and H-bonding interactions can planarize the oxidized layers, resulting in deeper packing and a shorter gallery distance.

### 3.3. Morphology and Elemental Analysis

**Figure 5:**
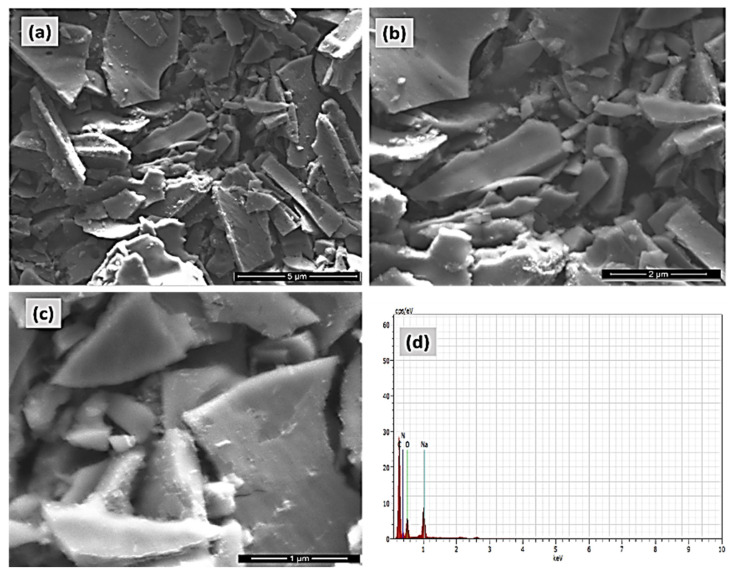
FE-SEM micrograph pictures of (**a**–**c**) Porous graphitic carbon nitride (P-GCN), and (**d**) EDAX and elemental analysis of P-GCN.

The morphology of P-GCN was examined using FE-SEM, as illustrated in Figure 5a–c. Figure 5a–c depicts the aggregated sheet-like morphology of polymeric porous materials of P-GCN products, which comprise of block-based textures and particles. The P-GCN used to have a characteristic dense and stacked lamellar structure of a few micrometers in size, whereas porous g-C_3_N_4_ agglomerated into non-uniform networks of tens of micrometers in width. The π-π stacking effect between layers and hydrogen bonding interactions mediated by oxygen-containing groups (-COOH, -OH) may promote network creation, which was confirmed by the low dense peak of the XRD results. The elemental composition of the P-GCN catalyst was investigated using EDX analysis, with the findings shown in Figure 5d.

**Figure 6:**
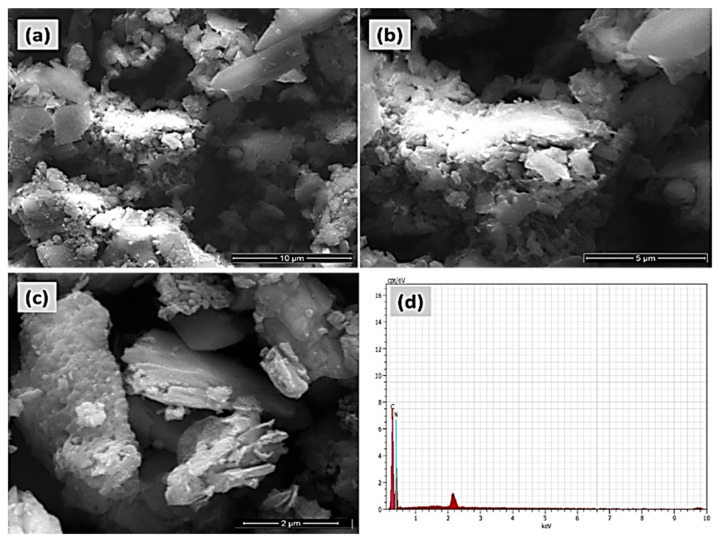
FE-SEM micrograph pictures of (**a**–**c**) covalent organic polymer (COP-N), and (**d**) EDAX elemental analysis of COP-N.

**Figure 7:**
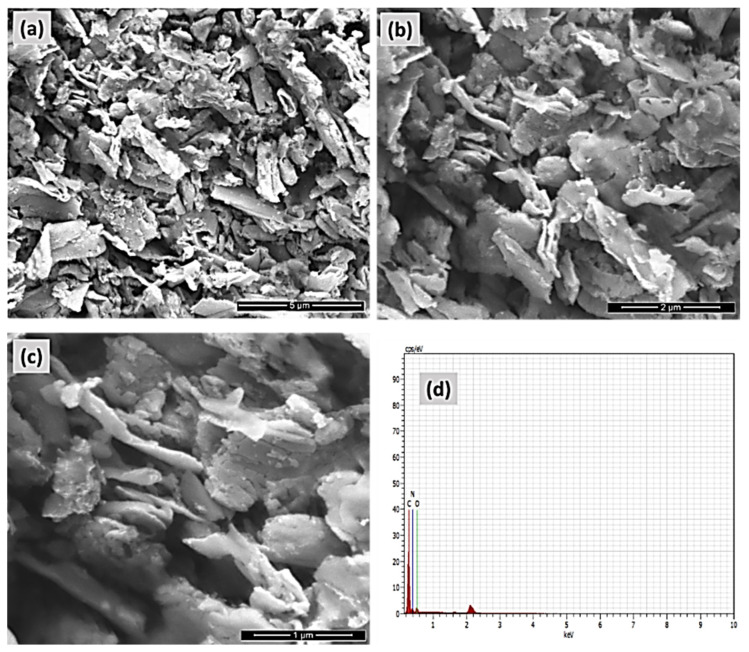
FE-SEM micrograph pictures of (**a**–**c**) covalent organic polymer (COP-N), and (**d**) EDAX elemental analysis of COP-N.

The FE-SEM and EDAX pictures of the COP-N are presented in Figure 6a–d. Similarly, the anthraquinone based covalent organic polymer (COP-A) FE-SEM and EDAX images are Showed in Figure 7a–d. Both the polymers exhibited larger surface areas and the pictures depict a layered-sheet morphology with dimensions of around 600 nm and thicknesses of about 100 nm. Particularly, the enhanced surface area helps to achieve the higher activity of the organic pollutant degradation. The distinctive metal free heterogeneous catalysts COP-N and COP-A reveal a porous polymer geometry and elemental composition of both materials, which was validated by EDAX analysis. The 2D layer-like morphology is more suitable for catalyst and dye pollutant interaction.

### 3.4. Optical Analysis

**Figure 8:**
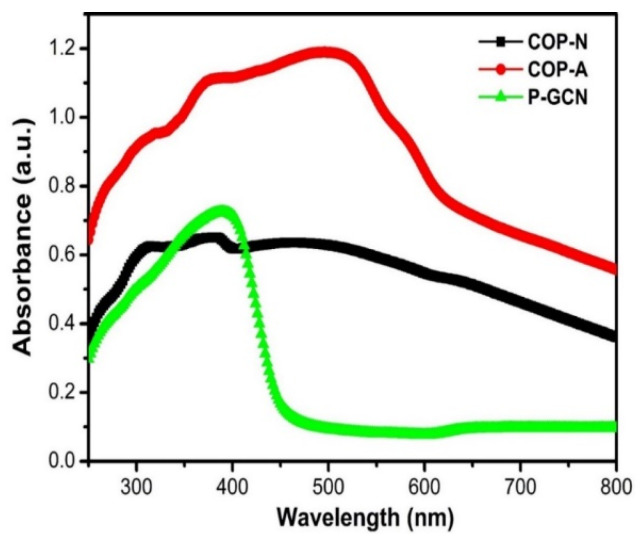
UV-visible spectra of COP-N, COP-A, and P-GCN.

The optical absorption of the three catalysts COP-N, COP-A, and P-GCN was studied. P-GCN catalyst absorbs UV over the visible range up to 450 nm, as illustrated in Figure 8. Band gap was determined from absorption values using a Tauc plot [40,41,42]. This result can be assigned to the intrinsic band gap of P-GCN (2.74 eV), which is slightly higher than B-GCN (2.70 eV). Because the absorption spectrum determines the color of a sample, the blue shift of the absorption edge corresponds to the color change from yellow of B-GCN to light yellow of P-GCN. Moreover, the COP-A showed a broad absorption peak which covered up to 600 nm in the visible region with a narrow band gap (~2 eV).

### 3.5. Surface Area Analysis

**Figure 9:**
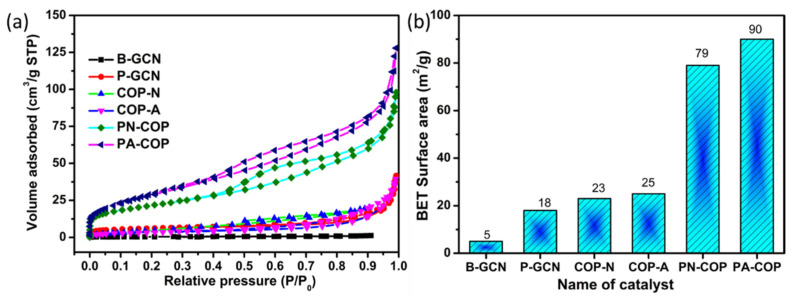
(**a**) N_2_ adsorption/desorption isotherms of the catalysts (**b**) histogram representing the surface area of the different photocatalysts.

The specific surface area is important for photocatalytic activity of a photocatalyst, because photocatalytic reactions occur on the surface of the photocatalyst. The N_2_ adsorption–desorption isotherms and BET bar diagrams of b-GCN, P-GCN, COP-N, COP-A, PN-GCN, and PA-GCN are compared in Figure 9a,b. The Brunauer–Emmett–Teller (BET) surface areas of B-GCN, P-GCN, COP-N, COP-A, PN-GCN, and PA-GCN were 5, 18, 23, 25, 79 and 90 m^2^/g, respectively The metal free catalyst of mesoprous structure of P-GCN exhibits a higher surface area than B-GCN. Similarly, the metal free composites of PN-GCN and PA-GCN obtained higher surface areas compared to the other catalysts. The high specific surface area stimulates us to investigate its potential applications in photocatalytic fields [43,44].

### 3.6. Photocatalytic Analysis

**Figure 10:**
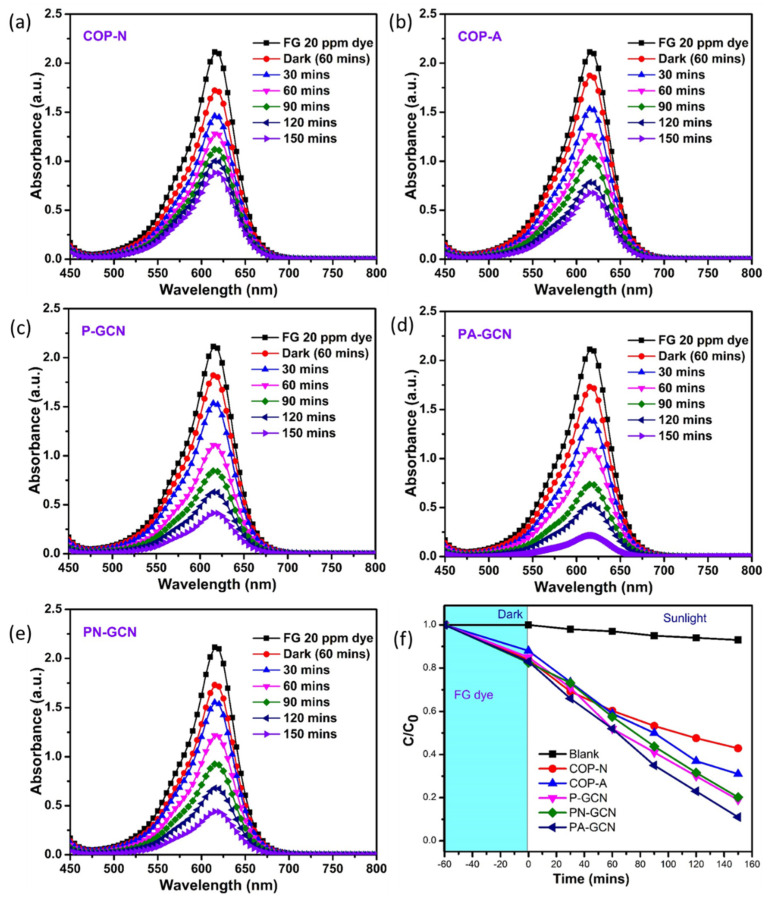
UV-Vis spectra of FG for the Metal free catalysts (**a**) COP-N, (**b**) COP-A, (**c**) P-GCN, (**d**) PA-GCN, (**e**) PN-GCN and (**f**) efficiency plot (C/C_0_) of FG.

Photocatalytic research and activity are influenced by surface area, visible light absorption, and blending substance. Metal free catalysts (COP-N, COP-A, P-GCN, PN-GCN, PA-GCN) were evaluated against organic pollutants using sunlight irradiation. Figure 10a–e depicts the UV–Visible spectra of FG dye in the presence of a catalyst treated for 120 min. The strongest absorption peaks were gradually lowered when the FG solution was handled, and the dramatic color dilution corroborated this concept. The degrading efficiencies of the COP-N, COP-A, P-GCN, PN-GCN, and PA-GCN were 57.07, 68.86, 80.66, 79.71, and 88.20%, respectively. The results clearly reveal that PA-GCN has the best degradation performance.

The efficiency plot (C/C_0_) of FG solution is represented in Figure 10f. The PA-GCN demonstrated the highest degradation ability and it is represented in the efficiency plot. The higher visible light absorption and lower recombination rates contribute to the production of a significant number of reactive oxygen species (ROS) [45]. 

**Figure 11:**
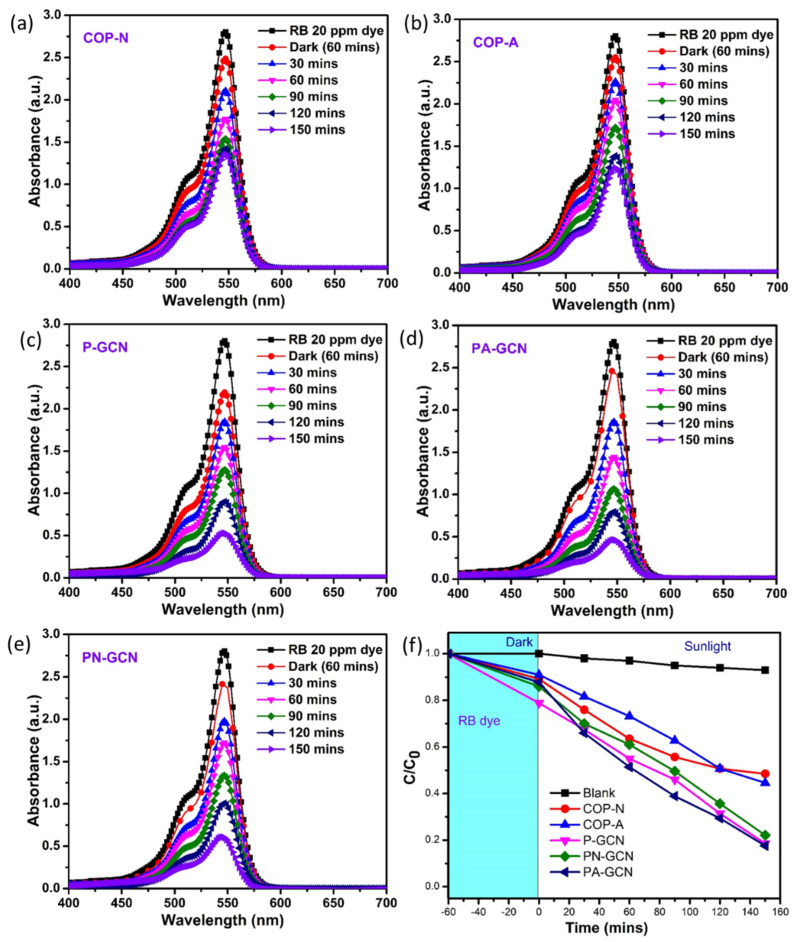
Degradation UV-Vis spectra of RB for the Metal free catalysts (**a**) COP-N, (**b**) COP-A, (**c**) P-GCN, (**d**) PA-GCN, (**e**) PN-GCN and (**f**) efficiency plot (C/C_0_) of RB.

Similarly, the photocatalytic investigations were studied for prepared nanocomposites against RB dye solution; the results are shown in Figure 11a–e. The COP-N, COP-A, P-GCN, PN-GCN, and PA-GCN catalysts showed the efficiencies of 57.07, 68.86, 80.66, 79.71, and 88.20%, respectively under identical conditions. The key enhancement in degrading efficiency is owed to the successful blending of COP in P-GCN, which increases the maximum light absorption, and has a lack of excited charge carrier recombination. The efficiency plot (C/C0) of RB solutions are depicted in Figure 11f. The rapid degradation of both FG and RB dyes illustrates synergetic effect of the PA-GCN catalyst. For comparison purposes, the k and R^2^ values are described in Table 1. Among the various concentrations, the C/C0 graph clearly demonstrates the maximal degradation ability of PA-GCN for both dye solutions.

**Figure 12:**
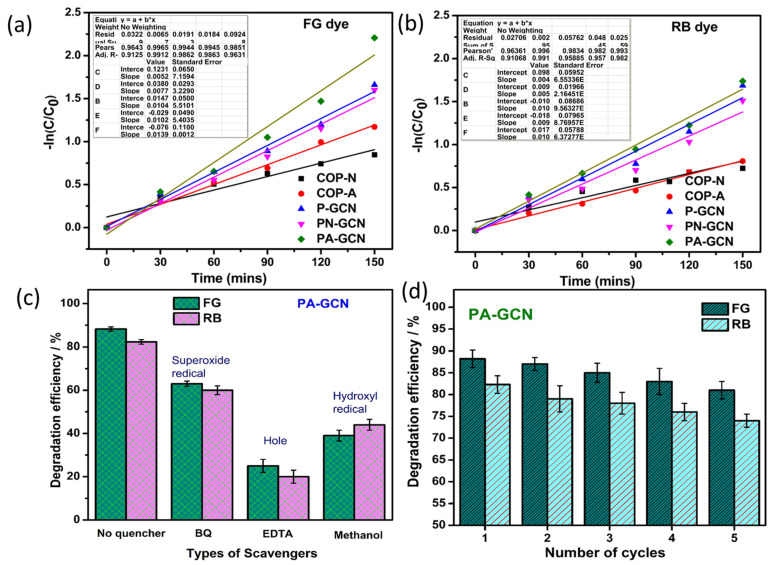
Degradation kinetic studies of (**a**) FG and (**b**) RB dye, (**c**) scavenger study, and (**d**) reusability of PA-GCN catalyst.

The rate constants for COP-N, COP-A, P-GCN, PN-GCN, and PA-GCN against FG dye are 0.011/min, 0.017/min, 0.023/min, 0.023/min, and 0.032/min, respectively. The kinetic plot is shown in Figure 12a. Further, the rate constants for COP-N, COP-A, P-GCN, PN-GCN, and PA-GCN against RB dye are 0.009/min, 0.012/min, 0.023/min, 0.020/min, and 0.024/min, respectively. The kinetic plot is shown in Figure 12b.

When exposed to visible light, the photoactive catalyst absorbs photons and converts them into electron–hole charge carriers. Generally, the catalyst makes three different types of ROS, and regulation on which ROS have the strongest effects during photodegradation is needed. The scavenger experiment assists in understanding the mechanism of dye photodegradation over the PA-GCN catalyst. EDTA, methanol and *p*-BQ are scavenger reagents of h^+^ scavenger, ·OH scavenger, and O_2_^−^ scavengers, respectively. These were added to the reagent to suppress the activity of h^+^, ·OH, and. O_2_^−^. Figure 12c depicts the percentage of scavengers that have degraded. Following the scavenger studies, the presence of EDTA and methanol shows major suppressing activity in the degradation of both dyes [46,47]. The structure and morphology are vital to the efficiency of degradation under sunlight. Here, the higher visible light absorption and surface-active sites are increased while incorporating the COP and P-GCN, which helps the enhancement of degradation efficiency of catalysts against FG and RB dye.

**Figure 13:**
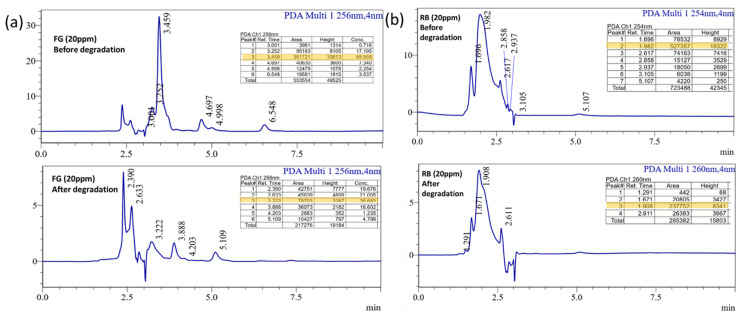
HPLC analysis of the (**a**) FG (**b**) RB dye solution of before and after the experiment.

The reusability of dye degradation catalysts after five reuses is shown in Figure 12d. Catalyst (powder form) renewal is often difficult, and catalysts compensate for the loss by renewing correctly. Finally, the degradation products of treated and untreated dye solutions are studied using the well-known standard technique of high-pressure liquid chromatography (HPLC). Figure 13a,b depicts the peaks at 3.4 (FG) and 1.9 (RB); retention time (RT) peaks are reduced due to the photocatalytic activity and this result confirmed that the bulky molecule degraded into simpler molecules [48]. In these results exhibits the area of the RT reduced after the photocatalytic experiment and its value highlighted in the Figure 13a,b. 

### 3.7. Photo Catalysis Mechanism

The photocatalytic mechanisms are as mentioned below,

(i) Production of excitons

PA-GCN + hν → h^+^ (VB) + e^−^ (CB) (3)

(ii) Hydroxyl radicals Generation

OH^−^ + h^+^ → OH· (4)

H_2_O + h^+^ → OH· + H^+^ (5)

(iii) Superoxide radicals Generation

O_2_ + e^−^ → O_2_·^−^ (6)

(iv) Degradation of dye molecules

Dye + OH· + O_2_·^−^ → Degradation products (7)

**Figure 14:**
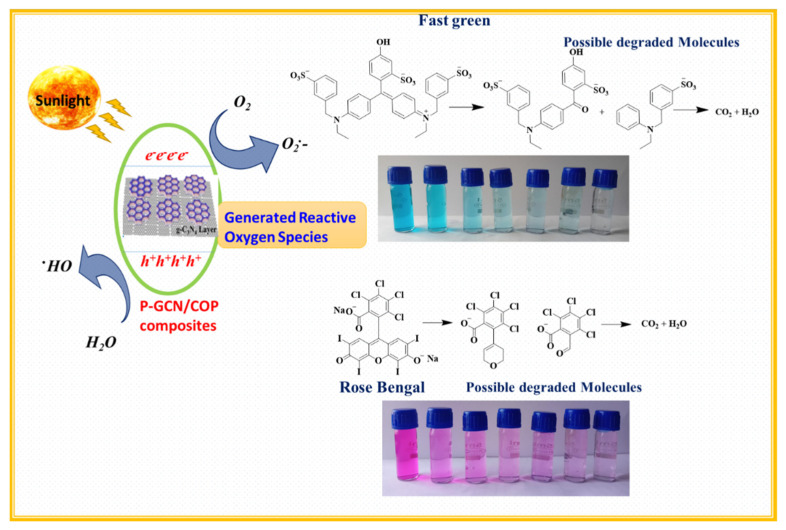
Schematic diagram mechanism of photocatalytic dye degradation.

In this current analysis, PA-GCN NPs show a higher percentage of efficiency than the NPs of COP-N, COP-A, P-GCN, and PA-GCN. The enhancement of catalyst behavior could be attributable to two basic factors: (i) higher light absorption and (ii) catalytic surface. Inhibiting photogenerated charge carrier recombination in PA-GCN helps to enrich percentage of degradation. In the COP-A, an electron-rich group near the Schiff base centers can stabilize the skeleton and build an electron donor–acceptor system with a triazine unit to alter the photoelectric characteristics and improve photocatalytic efficiency. Furthermore, the C=N bond coupling between the triazine and anthraquinone rings was used to create complete conjugated polymers with rapid charge-carrier mobility. In the skeleton, there are three distinct molecular donor–acceptor domains comprised of anthraquinone, phenyl units (electron donor), and the triazine ring (acceptor), demonstrating a photogenerated energy transfer cycle from higher to lower energy and increased intramolecular electron transfer. Further, electrons transfer from the HOMO orbitals of the electron-donor units to the LUMO orbitals of the electron-acceptor units in this photocatalytic process, thereby suppressing charge recombination. However, the employment of a single COP catalyst is often restricted by the low efficiency caused by the quick recombination of the photogenerated electron–hole pairs. One method for increasing photocatalytic efficiency is to build composite photocatalysts by combining COP-N and COP-A with P-GCN. The advantage of composite photocatalysts is the dramatically improved charge separation and transfer efficiency caused by the difference in band levels between COP and P-GCN. This results in the migration of excited electrons from one semiconductor’s higher CB (LUMO orbital) to another’s lower CB, as well as holes from one semiconductor’s higher VB (HOMO orbital) to the lower one. Attentively, the PA-GCN catalyst exhibited brilliant photocatalytic activity under sunlight. The schematic diagram mechanism of photocatalytic dye degradation shown in Figure 14.

## 4. Conclusions

In summary, the COP-N, COP-A, and P-GCN catalysts have been successfully synthesized by a simple and affordable thermal and chemical condensation approach and have been characterized using various characterization techniques. The FE-SEM images show metal free catalysts have a stacked lamellar structure. The photocatalytic performance of COP-N and COP-A were effectively improved when combined with P-GCN. The as-prepared catalyst is an excellent photoactive catalyst for the degradation of FG and RB dyes when exposed to visible sunlight. The PA-GCN catalyst demonstrated best photodegradation efficiency of 88.2% for FG and 82.3% for RB. It was determined that the rate constant value of PA-GCN (0.032/min) was almost three times higher than that of COP-N for FG degradation. Similarly, the rate constant value of PA-GCN (0.024/min) for RB degradation was much higher than that of COP-N. The increased photocatalytic activity is closely related to the successful blending of COP and P-GCN, and it plays a critical role in increasing greater light absorption, and increasing the formation of whole and hydroxyl radicals, which promote the activity and stability of the catalyst. The photodegradation results demonstrate the catalyst’s great stability even after five cycles.

## Figures and Tables

**Table 1:** Degradation parameters of COP-N, COP-A, P-GCN, PN-GCN, and PA-GCN against FG and RB dye solution.

S. No	Catalyst	Degradation Efficiency (%)	K (min^−1^)	R^2^
FG dye solution				
1	COP-N	57.07	0.011	0.91
2	COP-A	68.86	0.017	0.99
3	P-GCN	80.66	0.023	0.98
4	PN-GCN	79.71	0.023	0.98
5	PA-GCN	88.20	0.032	0.96
RB dye solution				
1	COP-N	51.4	0.009	0.91
2	COP-A	55.3	0.012	0.99
3	P-GCN	81.4	0.023	0.95
4	PN-GCN	77.8	0.020	0.95
5	PA-GCN	82.3	0.024	0.98

## Data Availability

Not applicable.
